# The Impact of Socioeconomic Status on Visual Acuity Changes in Schoolchildren: A One-Year Follow-Up

**DOI:** 10.3390/children11101226

**Published:** 2024-10-09

**Authors:** Alba Galdón, Núria Vila-Vidal, Mariam El Gharbi, Valldeflors Vinuela-Navarro, Joan Pérez-Corral, Núria Tomás, Laura Guisasola

**Affiliations:** 1 Visió Optometria i Salut, Department of Optics and Optometry, Universitat Politècnica de Catalunya, 08222 Terrassa, Spain; nuria.vila@upc.edu (N.V.-V.); mariam.el.gharbi@upc.edu (M.E.G.); valldeflors.vinuela@upc.edu (V.V.-N.); juan.enrique.perez@upc.edu (J.P.-C.); nuria.tomas@upc.edu (N.T.); laura.guisasola@upc.edu (L.G.); 2 Centre Universitari de la Visió, Universitat Politècnica de Catalunya, 08222 Terrassa, Spain

**Keywords:** children, visual acuity, socioeconomic status, myopia

## Abstract

(1) Background: Visual acuity (VA) is essential for children’s quality of life, and its relationship with socioeconomic status (SES) highlights disparities in healthcare. This study investigated the influence of SES on changes in schoolchildren’s VA over one year. (2) Methods: Initial examinations were conducted on 1822 children (8–10 years). Follow-up was performed on 804 of these children a year later. Uncorrected (UCVA) and presenting (PVA) distance VA were measured monocularly using a decimal Snellen chart. Very reduced UCVA (<0.5) was considered a proxy of myopia. (3) Results: The prevalence of initially very reduced UCVA (myopia) was similar in children with low and high SES (12.6% vs. 12.4%) (χ^2^; *p* = 0.153). After one year, the prevalence of very reduced UCVA increased to 14.1% in children with a low SES compared with 11.1% in children with a high SES (*p* = 0.001). Significant disparities related to SES were also found in PVA so that children with a low SES exhibited a greater reduction in PVA than children with a high SES (5.2% vs. 3.5%) (χ^2^; *p* = 0.004). (4) Conclusions: Children with a low SES showed an increase in reduced UCVA values over one year and a higher number of children with very reduced PVA compared with those with a high SES.

## 1. Introduction

The World Health Organization estimates that there are approximately one billion individuals worldwide who suffer from visual impairments that could have been prevented or have been left untreated, with a significant impact on individuals with a low SES [1]. Of these impairments, 43% are attributed to uncorrected refractive errors (myopia, astigmatism, and hyperopia), making them the leading cause of preventable visual disability in children [2]. The prevalence of significant refractive errors and the economic value associated with their correction present a global socioeconomic and public health issue [3].

In the context of educational equity, visual acuity (VA) and its relationship with socioeconomic status (SES) is a highly relevant topic [4]. VA represents the visual system’s ability to perceive fine details and distinguish objects at various distances; an adequate VA is essential in the daily lives of children [5]. SES, encompassing factors such as income, education, and occupation, is a key determinant of health, influencing access to healthcare services and overall living conditions [6].

Various genetic, social, and environmental factors influence the distribution of VA. Michael Marmot demonstrated that health disparities are not solely due to biological or genetic factors but are also influenced by social and economic conditions (unnatural causes) [7].

The lack of awareness about eye health is a significant issue, especially among families with a low SES, who may have greater difficulties accessing vision exam programs [8].

Numerous studies have shown that children from socioeconomically disadvantaged backgrounds are more likely to experience undetected vision problems, uncorrected refractive errors, and reduced visuomotor skills [9,10,11]. Several studies indicated that children with uncorrected refractive errors scored lower on various motor and cognitive tests [12,13,14,15].

These issues can negatively impact school readiness, cognitive performance, and future academic achievements, as they are linked to learning-related activities, such as writing [8]. Furthermore, reduced VA upon school entry has been associated with lower literacy levels, which has significant implications for children’s educational, health, and social interactions in the future [16,17].

Evaluating uncorrected visual acuity (UCVA) is crucial to determine whether children from lower socioeconomic backgrounds have worse vision compared with children from higher socioeconomic backgrounds, before considering the necessity of optical correction [8,18].

Reduced UCVA could be considered as a proxy for myopia, with various studies indicating that low UCVA in children is primarily due to myopia rather than astigmatism or hyperopia [19,20,21]. VA and refractive error, particularly myopia, have been shown to be closely related in children [19]. VA significantly impacts children’s academic performance and quality of life, with a minimum VA of 0.5 decimal often required to perform regular classroom tasks [22]. Myopic children struggle to see distant objects clearly, which can affect their academic performance and daily activities. Early correction of myopia is, therefore, crucial to preserve optimal visual performance and ensure adequate VA [23].

In East Asia, studies have linked high levels of parental education and income to a higher prevalence of myopia in their children [24]. In a study in Northern Ireland, schools with higher academic levels had a higher prevalence of myopia compared with schools with lower socioeconomic levels [25]. However, research into socioeconomic disparities among 6-year-old children in the Netherlands found that families with low incomes and low parental education had a higher prevalence of myopia than those with high incomes and education levels [26]. A Dutch cohort study examining children at ages 6 and 9 found that children from lower-income families and with lower maternal education had a higher incidence of myopia than children with higher incomes and higher maternal education levels [27].

The disparity in results highlights the complex interactions between socioeconomic status in children and the incidence of myopia (low VA) across different cultural and geographical contexts. Therefore, more research is needed to fully understand this relationship.

Presenting visual acuity (PVA) depends on families’ financial ability to acquire suitable glasses and their understanding of the importance of regular eye examinations to maintain updated optical correction (health literacy) [28]. Since optical correction is not a public service in Spain, it commonly requires private economic expenditure from individual families. It is important to note that PVA is affected both by lack of correction and by outdated optical correction. Both reasons can lead to significant economic implications, as the cost associated with proper optical correction is essential for maintaining a suitable quality of life.

Additionally, it has been observed that correcting vision problems could lead to improvements in reading [15,29]. Therefore, a comprehensive eye examination should be considered for children aged 8 to 10 years old, as effective treatment and intervention for any visual anomalies at this stage can significantly enhance their educational performance [30].

This study aimed to investigate how SES influences changes in VA among schoolchildren over a one-year period, including assessing children’s use of glasses and their annual updates. Disparities in eye health potentially associated with different socioeconomic levels were sought to be identified by examining both UCVA and PVA. All analyses were stratified according to sex in order to evaluate whether there are differences between males and females in VA stability.

## 2. Materials and Methods

### 2.1. Study Design

This is a longitudinal and prospective epidemiological study being conducted at the Centre Universitari de la Visió (CUV) in Terrassa (Barcelona, Spain).

In this study, the changes in VA over a one-year period were analyzed. Two controls were carried out, with an interval of one year between them.

### 2.2. Study Population

**Figure 1:**
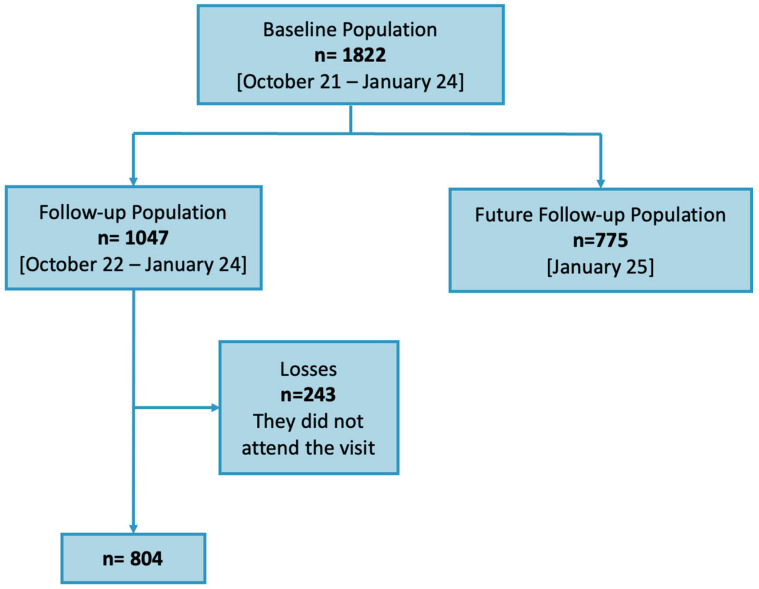
Flow chart showing the structure of the population sample for this study.

The initial population sample contained 1822 children from 16 primary schools in the city of Terrassa. The initial exam data were collected between October 2021 and January 2024. After one year, the 1047 children visited by January 2023 were invited to a follow-up visit between October 2022 and January 2024. A total of 804 attended. The age range of the children was between 8 and 10 years. The remaining 775 children will be evaluated in the next phase of this study (Figure 1).

The sample size was calculated with EpiData (EpiData Association, Odense. Denmark version 3.1) considering the reference population of 35,000 children of the city where this study was conducted and a confidence level of 95% and a 5% precision. According to such calculations, the minimum sample size required for this study was 246, which was lower than the 804 schoolchildren recruited in this study.

### 2.3. Data Collection

During both study visits, distance monocular UCVA was measured first, followed by PVA. A decimal Snellen chart was used for these measurements, placed at 6 m under photopic illumination, with a random letter order. This was measured using an optotype projector.

UCVA refers to uncorrected visual acuity, while PVA denotes the child’s habitual visual acuity. For children without any correction, their PVA will be equal to their UCVA. In contrast, for those who use optical correction, their PVA will represent their visual acuity with glasses or contact lenses.

Prior to the first study visit, a questionnaire was administered to the parents to gather information about the parents’ SES (educational level and employment status).

After the initial exam, the parents were provided with a brief report of their children’s visual abilities and difficulties with some guidelines and recommendations, if needed.

### 2.4. Definition of Variables

VA testing was conducted in both visits under two conditions: UCVA and PVA, without and with habitual optical correction, respectively. The UCVA and PVA results were recorded in decimal format and were divided into three categories: group 1—normal VA (>0.7); group 2—reduced VA (0.7 ≥VA≥0.5); and group 3—very reduced VA (<0.5).

The socioeconomic statuses of the child participants were obtained through the school classification into two groups: low SES (classified as high complexity) or high/average SES (unclassified). Criteria that relate to the students’ school environment form the basis of the classification. It includes the education and employment levels of the parents, the level of immigration, the number of new students, and the proportion of special educational support needs. These criteria are set by the Government of Catalunya [31].

This classification was verified in this study with the family questionnaire. The questionnaire considered the employment status and education of the parents at the time of this study.

The categories for SES included high SES—employed parents and parents with higher education or secondary education; low SES—unemployed parents and parents with primary education or no formal education.

### 2.5. Exclusion Criteria

This study excluded children with pathological ocular conditions, with disabilities that could complicate the vision exam, and whose parents did not give consent to participate in this study.

### 2.6. Statistical Analysis

For the statistical analysis, the data from the vision exam and questionnaires were analyzed with SPSS V.29.0.2.0 (20) (IBM SPSS, Istanbul, Turkey). Prior to the statistical analysis, the distribution of VA was assessed using the Shapiro–Wilk test, which revealed a non-normal distribution. Hence, non-parametric statistics were used to assess the relationship between VA and children’s SES.

The chi-square test was used to establish the association between VA and SES. Additionally, Mann–Whitney U tests were used to compare independent groups of VA. The differences in VA between the first and the follow-up visits were studied using Friedman tests. A *p*-value of less than or equal to 0.05 was considered significant.

## 3. Results

When analyzing the distribution of family SES, it was observed that in schools classified as having a low SES, there were approximately twice as many parents with basic education compared with parents in schools with an average/high SES. Table 1 shows that the fathers and mothers of children attending low-SES schools had an unemployment rate twice as high as that of those attending average/high-SES schools. These findings confirm the classification by the “Generalitat de Catalunya” government.

Given this confirmation of the school division, the official classification of high-SES and low-SES schools was used in the results.

### 3.1. Initial Visit

A total of 1822 children (49.9% male and 50.1% female) aged 8–9 years attended the first study visit. The average age of all participants was 8.80 (95% CI: 8.78–8.83) years old. A total of 802 (44%) participants were classified as having a low SES, with 52.6% males and 47.4% females, based on the categorization of the schools.

When the visual acuities of both eyes were compared, a Pearson’s r of 0.90 (UCVA) and 0.815 (PVA) was obtained (*p*-value < 0.001). These values indicate a high correlation between the VAs of both eyes. Therefore, future statistical analysis will only consider the VA of the right eye.

In the analysis of UCVA, 12.5% of schoolchildren had very reduced VA (<0.5), indicating possible myopia, with a significant improvement observed in PVA, which was 4.2%.

There were no statistically significant differences in UCVA and PVA by gender (UCVA: *p* = 0.419; PVA: *p* = 0.083) (Table 2).

The UCVA distribution did not show an association with family SES (*p* = 0.153), suggesting similar acuity at both socioeconomic levels. In contrast, a link between PVA and SES was found, indicating that more schoolchildren with reduced PVA were found in the low-SES group (χ^2^; *p* = 0.004). This statistical significance was observed in VA groups 1 and 2 (Mann–Whitney U; Z= −2.506; *p* = 0.012) (Table 3).

### 3.2. Follow Up

A follow-up exam was conducted on 804 children who had an average age of 9.80 (95% CI: 9.77–9.90). There were 390 (48.4%) males and 414 (51.6%) females, of whom 270 had a low SES (49.3% males vs. 50.7% females) based on the school category classification.

The results reveal a statistically significant difference in UCVA after one year (Friedman; *p* = 0.030). A substantial rise in students with reduced and very reduced UCVA was observed in low-SES schools, indicating a higher incidence of proxy myopia in such schools (Friedman; *p* < 0.001). High-SES schools did not show any link with UCVA (Friedman; *p* = 0.837) (Table 4).

When considering gender, children’s UCVA did not show any significant differences, suggesting no differences in the presence of proxy myopic refractive error between males and females. This was the case for both low- and high-SES schoolchildren at the first visit (χ^2^; *p*= 0.393 for males and *p* = 0.140 for females) as well as at the follow-up visit (χ^2^; *p* = 0.914 for males and *p* = 0.951 for females) (Table 4).

**Figure 2:**
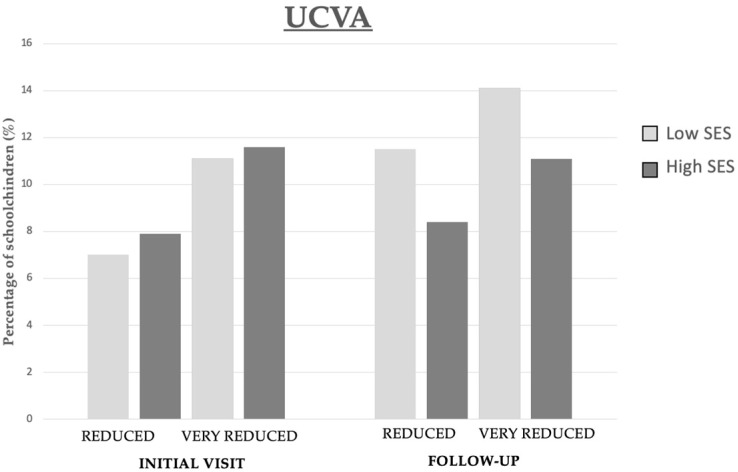
Prevalence of reduced and very reduced UCVA in relation to SES of schoolchildren at the initial visit and one-year follow-up.

As illustrated in Figure 2, no significant increase was observed in the prevalence of reduced or very reduced UCVA among high-SES schoolchildren after one year. However, there was a notable rise in the proportion of low-SES schoolchildren presenting with reduced UCVA (4.5%) and very reduced UCVA (4.0%) at the follow-up. This trend suggests a potential disparity in the progression of refractive error across different socioeconomic levels.

The one-year PVA follow-up did not show significant changes (Friedman; *p* = 0.226) (Table 5). The high-SES group did not show any improvement in PVA since the beginning of the study (Friedman; *p* = 0.827). As for the low-SES group, students needing optical correction based on reduced VA increased slightly. The rate rose from 13.7% to 17% in one year (Friedman; *p* = 0.116) (Table 5).

Gender differences were not found in either the low- or high-SES groups at the first visit (χ^2^; *p* = 0.855 for males and *p* = 0.388 for females). In contrast, significant differences were observed in females during the follow-up (χ^2^; *p* = 0.01). More female students had reduced PVA (15.6%) in the low-SES group than in the high-SES group (6.8%), but no differences were found in the male gender (χ^2^; *p* = 0.633).

**Figure 3:**
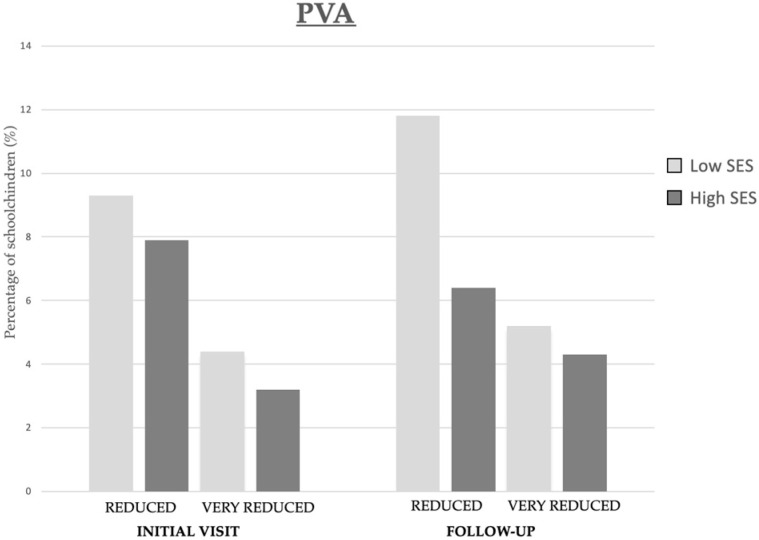
Prevalence of reduced and very reduced PVA in relation to SES of schoolchildren at the initial visit and one-year follow-up.

As shown in Figure 3, no significant changes were detected after one year of follow-up in reduced and very reduced PVA. However, a slight increase was observed in the proportion of low-SES schoolchildren with reduced (2.5%) and very reduced PVA (0.8%).

In relation to the use of optical correction, we observed a slight non-statistically significant increase in the number of students using them. In the high-SES group, the proportion of children wearing optical correction increased from 12% to 16.3% (χ^2^; *p* = 0.225). Similarly, in the low-SES group, an increase from 10.3% to 14.8% in the use of optical correction was observed (χ^2^; *p* = 0.115) (Table 6).

During the first visit, no differences were observed between male students, neither in SES nor in optical correction use (χ^2^; *p* = 0.604). However, there was a difference between females and SES (χ^2^; *p* = 0.018). It was observed that females wore optical correction at a rate of only 5.9%, whereas males wore optical correction at a rate of 14.8% in the low-SES group (Table 6).

In the follow-up, there were no significant differences in both genders and the use of optical correction (Friedman; *p* = 0.667 for males and *p* = 0.669 for females) (Table 6).

## 4. Discussion

This study assessed the changes in VA in a school population over a period of one year. The possible association between the children’s SES and VA was examined. To this end, two conditions were considered: UCVA, which evaluated the visual capacity without the use of optical correction and was considered a good indicator of the presence of myopia, and PVA, which included the ability to acquire optical correction and health literacy, which provided the necessary knowledge to assess the need for optical correction, its updating, and its acquisition.

The distribution of UCVA and PVA during the initial visit in 1822 children was analyzed. It was found that 12.5% (n = 227) of the study population had a UCVA below 0.5, indicating refractive errors, possibly myopia. Myopia has a greater impact on children’s VA than hyperopia and astigmatism, resulting in very reduced VA (<0.5) [19,20,21].

The prevalence of myopia is very similar to that obtained in a study conducted in Rotterdam (The Netherlands), where 11.5% of children of the same age (9 years old) were recorded [32]. It is also consistent with findings from another study conducted in Spain, which reported a prevalence of 11.1% in children of a similar age [33].

Regarding very reduced PVA, it was found that 4.2% (n = 77) of students were not adequately corrected, as their PVA was less than 0.5. This level of VA may hinder their academic performance and achievement, as their learning experience is challenged due to reduced vision.

Firstly, at the initial visit, no relationship was found between UCVA and SES in the 1822 examined children. When studying possible differences in UCVA between low- and high-SES 8–9-year-old children with refractive error, no statistically significant differences were found, thus reinforcing Philipp et al.’s results, which found no relationship between myopia and SES [34].

Similarly, no significant differences were observed in relation to gender, unlike the results of Yang et al., which did associate low SES with lower UCVA in 7–9-year-old children, finding a higher number of females with poor UCVA (*p* < 0.05) [21].

These results suggest that SES does not have a direct impact on children’s UCVA in this age range. The prevalence of initially very reduced UCVA (myopia) was similar in children with low and high SES (12.6% vs. 12.4%) (χ^2^; *p* = 0.153). Future research should explore other factors, such as genetics, living environment, or visual habits in Spain, and should also include longer study periods to provide a more comprehensive understanding of these influences.

In contrast, when assessing PVA in the initial visit, statistically significant SES differences were found. A higher percentage of children from low-SES backgrounds had reduced PVA. The rates were 12.3% for reduced PVA and 5.2% for very reduced PVA in children from low-SES backgrounds, while the percentages were 8.5% and 3.5% in children from high-SES backgrounds, respectively.

These findings suggest that children from low-SES backgrounds may face a challenge in accessing proper optical correction and adequate eye care [8]. Lack of funds, limited access, low health literacy level, and privatized eye care could all contribute to these disparities.

Parents may not be aware of how important it is for their children to undergo regular eye vision examinations [28,35]. In the case of Spain, the country where this study was conducted, adequate optical corrections are a private matter. This poses challenges for parents with low health literacy to understand the need to correct vision problems at these ages. A study in Ireland reported over half of parents did not believe myopia was a health risk to their children. Only 14% of people showed concern about its development [36], demonstrating the parents’ limited knowledge of the importance of proper PVA.

Secondly, an evaluation of the findings was carried out during the one-year follow-up. Children from low-SES families had higher rates of reduced and very reduced UCVA (proxy of myopia) compared with children from high-SES families. The prevalence of very reduced UCVA increased to 14.1% in children with a low SES compared with 11.1% in children with a high SES (*p* = 0.001). This means there was an increasing incidence of refractive errors for low-SES children.

Two studies support our findings and delve into the socioeconomic differences among Dutch children. One study found that 6-year-old children from families with lower incomes and less education had more myopia. Their visual habits, specifically increased screen time, were related [26]. Another cohort study identified a higher incidence of myopia in children between the ages of 6 and 9 among those with lower incomes and mothers with lower levels of education [27].

Conversely, studies conducted in other regions of the world contradict these findings. Recent studies in Asia found that myopia begins and progresses during school years and is linked to higher parental incomes and more education [37]. A study in northern India showed that myopia was more common in children from families with higher SES and children attending private schools. This was likely due to the extra time they spent at home reading, writing, watching TV, or using electronic devices [24]. Also, researchers found that high-SES schools had a 2.5% higher rate of myopia in Northern Ireland [25].

However, most recent studies on refractive error and SES in Europe have focused on adults. Their results indicate that higher education is associated with increased refractive error, especially myopia [38,39,40,41].

In short, this study’s results agree with studies in the Netherlands showing a link between low SES and the rate of proxy myopia in children [26]. This study also found a higher frequency of myopia in children from families with low incomes (OR: 2.62; 95% CI: 1.8 to 3.74) and low maternal education (OR: 2.27; 95% CI: 1.57 to 3.28). This controversy shows the multifactorial etiology of myopia.

The association between gender and eye problems in children is controversial. Several studies report that females have higher myopia rates [38,42,43]. Conversely, other studies do not support this correlation, as in our study, since no differences were found between gender and UCVA [44,45,46].

This study’s findings indicated no statistically significant changes in PVA within a year. Thus, children from low-SES families continued to have higher rates of reduced PVA compared with those from high-SES families. This has a significant impact on academic performance, as reduced VA is associated with lower academic achievement [11]. Additionally, it has been demonstrated that diminished PVA in children is linked to slower development of literacy skills [16].

When analyzing gender differences after one year, it was found that females from low-SES backgrounds had a higher percentage of very reduced PVA (VA < 0.5), indicating that they were worse well-corrected compared with females from high-SES backgrounds.

This could be due to the maturation process in females being earlier than in males, occurring before the age of 8 in females and 9 in males [47,48]. Therefore, growth may lead them to develop myopia at a younger age than males.

While the results found could be an indicator of gender discrimination, we do not have evidence directly pointing to this cause. A plausible explanation is that parents may be giving similarly limited attention to both sons and daughters, but the impact is greater in girls due to the earlier onset of myopia.

In addition to this potential increase in myopia, the lack of financial resources in families from low-SES backgrounds impedes the ability to afford regular eye examinations and updated optical corrections [8]. This lack of access contributes to a higher prevalence of very reduced PVA among females from low-SES backgrounds.

During one year of follow-up, despite the visual guidelines offered to parents, the use of optical correction did not change significantly. Parents were advised to update their children’s optical prescription, if necessary, to limit the use of electronic devices and to encourage outdoor activities. Furthermore, it is important to take regular visual breaks and to promote frequent blinking in order to prevent visual fatigue.

In contrast to our findings, a study in Turkey also provided eye health advice [49]. This advice increased optical correction usage within a year to a greater extent than observed in our study. This finding raises questions about our current interventions. It shows the need for better long-term strategies in managing refractive errors [50].

In the follow-up, losses in the low-SES group were primarily due to children’s school absenteeism, which prevented them from attending scheduled follow-up visits. To address this issue, it is crucial to implement strategies that allow for greater flexibility in scheduling appointments and to raise awareness among parents about the importance of their children attending these check-ups.

In conclusion, the results of this study indicate that SES is a relevant factor in children’s vision care. Additionally, they highlight the need to ensure access to visual care for all children, as it was observed that those from low-income families require greater attention and better visual conditions.

## 5. Conclusions

In the cohort studied, a significant impact of SES on VA was found. Children from low-income families were more likely to have uncorrected refractive errors based on reduced VA. This is important, as it can hinder children’s academic performance and overall quality of life. Given the considerable economic burden associated with myopia, it is imperative to establish preventive strategies for children of all ages.

This study highlights the need to improve access to and knowledge of optometric and ophthalmological care in Spain, especially in children from low-income families. Overcoming obstacles, such as a lack of financial resources and limited awareness among parents about the importance of regular visual exams, is essential to ensure that all children receive appropriate visual care.

In conclusion, there have been few studies analyzing SES in children populations and its relationship with vision, resulting in little data and few results to review or analyze. Future research should focus on studying refractive errors and PVA in economically disadvantaged children.

Future research directions should be considered. While visual acuity is an essential factor, it is important to recognize that other visual functions beyond refraction, such as axial length, can significantly influence outcomes. Specifically, binocular vision and accommodative function are critical aspects that warrant further investigation. By exploring these additional visual parameters, future studies can provide a more comprehensive understanding of visual performance and its implications for adequate eye health.

## 6. Limitations

An important limitation was the difficulty in tracking low-SES participants, as many did not attend the follow-up visit a year later, potentially biasing group representation. Additionally, the evaluation focused only on visual acuity (VA) without considering refractive error. While most uncorrected refractive errors affecting VA at ages 8–9 are myopic, not all cases are solely due to this condition.

## Figures and Tables

**Table 1:** Distribution of schools based on families’ socioeconomic status (SES).

	Employment Status						Educational Level					
	Low-SES Schools		High-SES Schools				Low-SES Schools		High-SES Schools			
	Employed n (%)	Unemployed n (%)	Employed n (%)	Unemployed n (%)	Missing n	Total n	High Education n (%)	Basic Education n (%)	High Education n (%)	Basic Education n (%)	Missing n	Total n
Fathers	387 (70.6)	161 (29.4)	719 (87.7)	101 (12.3)	454	1822	320 (57.1)	240 (42.9)	656 (78.9)	175 (21.1)	431	1822
Mothers	221 (37.6)	367 (62.4)	565 (66.5)	285 (33.5)	384	1822	380 (65.4)	201 (34.6)	707 (82.2)	154 (17.8)	380	1822

**Table 2:** Distribution of uncorrected visual acuity (UCVA) and presenting visual acuity (PVA) in the initial visit sample regarding sex.

VA Categorization	UCVA				PVA			
	Males n (%)	Females n (%)	Total n (%)	*p*-Value Test χ^2^	Males n (%)	Females n (%)	Total n (%)	*p*-Value Test χ^2^
				0.419				0.083
>0.7 (normal)	722 (79.4)	702 (76.9)	1424 (78.1)		788 (50.5)	772 (49.5)	1560 (85.6)	
0.7 ≥VA≥0.5 (reduced)	79 (8.7)	92 (10.1)	171 (9.4)		79 (8.7)	106 (11.6)	185 (10.2)	
<0.5 (very reduced)	108 (11.9)	119 (13.0)	227 (12.5)		42 (4.6)	35 (3.7)	77 (4.2)	
Total	909	913	1822		909	913	1822	

**Table 3:** First visit distribution of uncorrected visual acuity (UCVA) and presenting visual acuity (PVA) according to school children’s socioeconomic status (SES).

		UCVA			PVA			
		High SES n (%)	Low SES n (%)	*p*-Value Test χ^2^	High SES n (%)	Low SES n (%)	*p*-Value Test χ^2^	*p*-Value Mann–Whitney U
				0.153			0.004	
VA	Group 1 (>0.7; normal)	810 (79.4)	614 (76.6)		898 (88.0)	662 (82.5)		G1–G3 0.052
	Group 2 (0.7 ≥VA≥0.5; reduced)	84 (8.2)	87 (10.8)		86 (8.5)	99 (12.3)		G1–G2 0.012
	Group 3 (<0.5; very reduced)	126 (12.4)	101 (12.6)		36 (3.5)	41 (5.2)		G2–G3 0.283
Total		1020	802		1020	802		

**Table 4:** Distribution of uncorrected visual acuity (UCVA) after one-year follow-up among different socioeconomic levels regarding gender.

		High SES							Low SES							*p*-Value Total
		Initial Visit			Follow-Up			*p*-Value	Initial Visit			Follow-Up			*p*-Value	
		Males n (%)	Females n (%)	Total n (%)	Males n (%)	Females n (%)	Total n (%)		Males n (%)	Females n (%)	Total n (%)	Males n (%)	Females n (%)	Total n (%)		
						0.837							<0.001	0.030
UCVA	>0.7 (normal)	206 (80.5)	224 (80.6)	429 (80.5)	211 (82.4)	219 (78.8)	430 (80.5)		111 (82.2)	110 (81.6)	221 (81.9)	105 (77.8)	96 (71.1)	201 (74.4)		
	(0.7 ≥VA≥0.5 (reduced)	17 (6.6)	25 (9.0)	42 (7.9)	19 (7.4)	26 (9.4)	45 (8.4)		8 (5.9)	11 (8.1)	19 (7.0)	10 (7.4)	21 (15.6)	31 (11.5)		
	<0.5 (very reduced)	33 (12.9)	29 (10.4)	63 (11.6)	26 (10.2)	33 (11.9)	59 (11.1)		16 (11.9)	14 (10.3)	31 (11.1)	20 (14.8)	18 (13.3)	38 (14.1)		
	Total	256	278	534	256	278	534		135	135	270	135	135	270		

**Table 5:** Distribution of presenting visual acuity (PVA) in the initial visit and after one-year follow-up among schools of different socioeconomic levels regarding gender.

		High SES							Low SES							*p*-Value Total
		Initial Visit			Follow-Up			*p*-Value	Initial Visit			Follow-Up			*p*-Value	
		Males n (%)	Females n (%)	Total n (%)	Males n (%)	Females n (%)	Total n (%)		Males n (%)	Females n (%)	Total n (%)	Males n (%)	Females n (%)	Total n (%)		
								0.827							0.116	0.226
PVA	>0.7 (normal)	228 (89.1)	247 (88.8)	474 (88.9)	228 (89.1)	249 (89.6)	477 (89.3)		118 (87.4)	115 (85.3)	233 (86.3)	116 (85.9)	108 (80.0)	224 (83.0)		
	(0.7 ≥VA≥0.5 (reduced)	17 (6.6)	25 (9.0)	42 (7.9)	15 (5.9)	19 (6.8)	34 (6.4)		11 (8.7)	14 (10.3)	25 (9.3)	11 (8.1)	21 (15.6)	32 (11.8)		
	<0.5 (very reduced)	11 (4.3)	6 (2.2)	18 (3.2)	13 (5.1)	10 (3.6)	23 (4.3)		6 (4.4)	6 (4.4)	12 (4.4)	8 (5.9)	6 (4.4)	14 (5.2)		
	Total	256	278	534	256	278	534		135	135	270	135	135	270		

**Table 6:** Distribution of optical correction in the initial visit and after one-year follow-up between schools of different socioeconomic levels regarding gender.

	High SES						Low SES					
	Initial Visit			Follow-Up			Initial Visit			Follow-Up		
	Males n (%)	Females n (%)	Total n (%)	Males n (%)	Females n (%)	Total n (%)	Males n (%)	Females n (%)	Total n (%)	Males n (%)	Females n (%)	Total n (%)
No optical correction	225 (87.9)	245 (88.1)	470 (88.0)	218 (85.1)	229 (82.4)	447 (83.7)	115 (85.2)	127 (94.1)	242 (89.7)	115 (85.2)	115 (85.2)	230 (85.2)
Optical correction	31 (12.1)	33 (11.9)	64 (12.0)	38 (14.9)	49 (17.6)	87 (16.3)	20 (14.8)	8 (5.9)	28 (10.3)	20 (14.8)	20 (14.8)	40 (14.8)
Total	256	278	534	256	278	534	135	135	270	135	135	270

## Data Availability

The data presented in this study are available upon request from the corresponding author due to ethical reasons.
